# Infection and risk of psoriasis and atopic dermatitis: A systematic review and meta-analysis

**DOI:** 10.1016/j.jdin.2025.12.007

**Published:** 2025-12-26

**Authors:** Zhiru Zhou, Xu Yao, Juan Su, Minxue Shen, Hervé Bachelez, Xiang Chen, Yi Xiao

**Affiliations:** a Department of Dermatology, Xiangya Hospital, Central South University, Changsha, Hunan, P.R. China; b Hunan Engineering Research Center of Skin Health and Disease, Xiangya Hospital, Central South University, Changsha, Hunan, P.R. China; c Hunan Key Laboratory of Skin Cancer and Psoriasis, Xiangya Hospital, Central South University, Changsha, Hunan, P.R. China; d National Clinical Research Center for Geriatric Disorders (Xiangya Hospital), Central South University, Changsha, Hunan, P.R. China; e Hospital for Skin Diseases, Institute of Dermatology, Chinese Academy of Medical Sciences and Peking Union Medical College, Nanjing, China; f Department of Social Medicine and Health Management, Central South University, Changsha, Hunan, P.R. China; g Department of Dermatology, Hopital Saint-Louis APHP, Paris Cité University, Paris, France

**Keywords:** atopic dermatitis, infection, meta-analysis, psoriasis, systematic review

*To the Editor:* Psoriasis and atopic dermatitis (AD) are common inflammatory diseases, characterized by chronic course and no known cure, highlighting the need to identify underlying etiology.^1^ Streptococcal pharyngitis is a well-established trigger of guttate psoriasis, and *Staphylococcus aureus* colonization contributes to AD progression.^2^^,^^3^ Moreover, the SARS-CoV-2 pandemic highlighted previously overlooked infectious triggers of immune dysregulation potentially linked to dermatological diseases.^4^ However, the impact of broader infections on the incidence of these diseases remains inconclusive. Therefore, we conducted a systematic review and meta-analysis to assess the relationship between infections and psoriasis or AD incidence.

Our review was registered in PROSPERO (CRD42023478691) following PRISMA and MOOSE guidelines (Supplementary Tables I and II, Appendix 1, available via Mendeley at https://data.mendeley.com/datasets/b5fvbd9djz/1). We searched PubMed, Web of Science, and Embase from inception to September 2025 for cohort studies examining infections and disease onset. Subgroup analysis stratified infections by type and site, while sensitivity analysis included studies using Cox proportional hazards model.

A total of 29 cohort studies (17 on psoriasis, 12 on AD) were included, encompassing 31,766,971 participants (Supplementary Fig 1, Table III, available via Mendeley at https://data.mendeley.com/datasets/b5fvbd9djz/1). Included studies were generally of high quality based on the Newcastle-Ottawa scale (Supplementary Table IV, available via Mendeley at https://data.mendeley.com/datasets/b5fvbd9djz/1). Infection was associated with a 64% increased risk of psoriasis (HR = 1.64, 95% CI: 1.32-2.04, *P* < .001), and a 23% increased risk of AD (HR = 1.23, 95% CI: 1.17-1.31, *P* < .001) (Supplementary Figs 2 and 3, available via Mendeley at https://data.mendeley.com/datasets/b5fvbd9djz/1). Subgroup analysis by infection type (Fig 1, Supplementary Figs 4 and 5, available via Mendeley at https://data.mendeley.com/datasets/b5fvbd9djz/1) revealed that viral (HR = 1.67), bacterial (HR = 1.40), and parasitic infection (HR = 3.03) were associated with psoriasis, while viral (HR = 1.18) and bacterial infection (HR = 1.50) were linked to AD. For infection site (Fig 2, Supplementary Figs 6 and 7, available via Mendeley at https://data.mendeley.com/datasets/b5fvbd9djz/1), psoriasis risk was elevated with skin (HR = 2.17) and respiratory tract infection (HR = 1.41), while AD incidence was linked to skin (HR = 1.76), respiratory tract (HR = 1.26), and gastrointestinal tract infection (HR = 1.42). Sensitivity analysis confirmed stable results (Supplementary Figs 8 and 9, available via Mendeley at https://data.mendeley.com/datasets/b5fvbd9djz/1), and the Egger test showed no evidence of publication bias.

**Fig 1:**
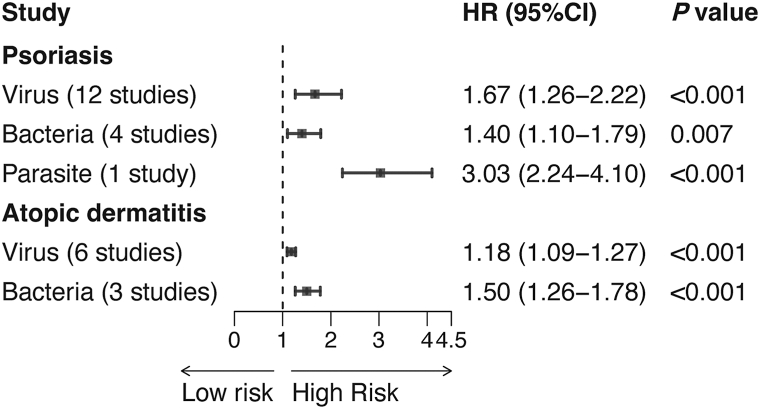
Psoriasis and atopic dermatitis. Summary of different types of infection and the incidence of psoriasis and atopic dermatitis. Data are presented as pooled hazard ratios (HRs) with 95% confidence intervals, calculated using random-effects models. Odds ratios and risk ratios from original studies were converted to HRs. Heterogeneity was assessed using I^2^ statistics and Cochran's Q-test. Detailed forest plots are shown in Supplementary Figs 4 and 5, available via Mendeley at https://data.mendeley.com/datasets/b5fvbd9djz/1. *CI*, Confidence interval; *HR*, hazard ratio.

**Fig 2:**
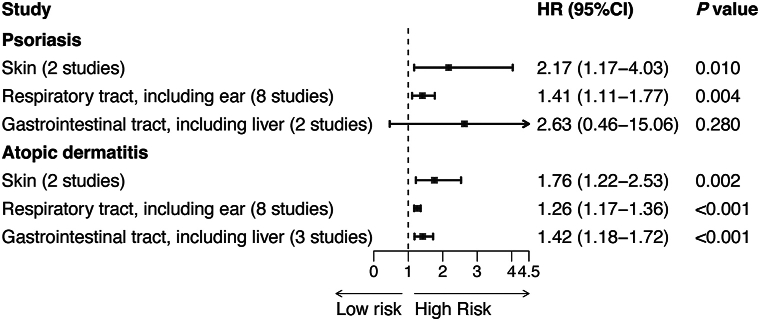
Psoriasis and atopic dermatitis. Summary of different sites of infection and the incidence of psoriasis and atopic dermatitis. Data are presented as pooled hazard ratios (HRs) with 95% confidence intervals. Statistical methods are as described in Fig 1. Detailed forest plots are shown in Supplementary Figs 6 and 7, available via Mendeley at https://data.mendeley.com/datasets/b5fvbd9djz/1.

Several limitations should be noted. First, although a random-effects model was applied to address heterogeneity, substantial variability remained, which may be explained by differences in infection definitions, study periods, or unknown disease etiology. Moreover, the observational design cannot establish causality, warranting further studies including Mendelian randomization or cytological experiments to strengthen causal inference. Additionally, the absence of cohort data for fungal infections in either psoriasis or AD limits generalizability and calls for high-quality cohort studies.

Despite limitations, our study provides epidemiological evidence that infection history should be considered a risk factor for both diseases. Broad infections extending beyond specific pathogens may act as a hit for chronic inflammation and immune stress in disease progression.^5^ Current clinical guidelines fail to adequately address infection-related risks, indicating the need for more comprehensive risk stratification strategies. Clinically, for individuals at high risk or with family history of disease, assessing infection history, controlling infections promptly, and modulating excessive immune response are essential. In patients with psoriasis or AD, especially on biological therapy, close infection monitoring is crucial to prevent disease flares. Further research should explore mechanistic links between systemic infections and immune dysregulation for targeted prevention strategies.

## Conflicts of interest

Xiao serves as Councilor of the International Psoriasis Council (IPC), member of the International Society of Atopic Dermatitis (ISAD), Associate Editor of the Journal of Investigative Dermatology (JID), and member of the Large Database Workgroup of the Journal of the American Academy of Dermatology (JAAD).
